# Developing a Digital Solution for Dengue Through Epihack: Qualitative Evaluation Study of a Five-Day Health Hackathon in Sri Lanka

**DOI:** 10.2196/11555

**Published:** 2019-08-29

**Authors:** Chitra Panchapakesan, Anita Sheldenkar, Prasad Wimalaratne, Ruwan Wijayamuni, May Oo Lwin

**Affiliations:** 1 Wee Kim Wee School of Communication and Information Nanyang Technological University Singapore Singapore; 2 School of Computing University of Colombo Colombo Sri Lanka; 3 Colombo Municipal Council Colombo Sri Lanka

**Keywords:** Epihack, civic engagement, dengue, digital epidemiology, participatory surveillance, participatory epidemiology, participatory design, workshop

## Abstract

**Background:**

Dengue is a mosquito-borne viral disease that has increasingly affected Sri Lanka in recent years. To address this issue, dengue surveillance through increasingly prevalent digital surveillance applications has been suggested for use by health authorities and the general public. Epihack Sri Lanka was a 5-day hackathon event organized to develop a digital dengue surveillance tool.

**Objective:**

The goal of the research was to examine the effectiveness of a collaborative hackathon that brought together information technology (IT) and health experts from around the globe to develop a solution to the dengue pandemic in Sri Lanka.

**Methods:**

Ethnographic observation and qualitative informal interviews were conducted with 58 attendees from 11 countries over the 5-day Epihack to identify the main factors that influence a collaborative hackathon. Interviews were transcribed and coded based on grounded theory.

**Results:**

Three major themes were identified during the Epihack Sri Lanka event: engagement, communication, and current disease environment. Unlike other hackathons, Epihack had no winners or prizes and was collaborative rather than competitive, which worked well in formulating a variety of ideas and bringing together volunteers with a sense of civic duty to improve public health. Having health and IT experts work together concurrently was received positively and considered highly beneficial to the development of the product. Participants were overall very satisfied with the event, although they thought it could have been longer. Communication issues and cultural differences were observed but continued to decrease as the event progressed. This was found to be extremely important to the efficiency of the event, which highlighted the benefit of team-bonding exercises. Bringing expert knowledge and examples of systems from around the world benefited the creation of new ideas. However, developing a system that can adapt and cater to the local disease environment is important in successfully developing the concepts.

**Conclusions:**

Epihack Sri Lanka was successful in bringing together health and IT experts to develop a digital solution for dengue surveillance. The collaborative format achieved a variety of fruitful ideas and may lead to more hackathons working in this way in the future. Good communication, participant engagement, and stakeholder interest with adaptation of ideas to complement the current environment are vital to achieve the goals of the event.

## Introduction

Dengue is a mosquito-borne viral disease that globally affects an estimated 390 million people each year [1]. In 2013, dengue was estimated to be responsible for 1.14 million disability-adjusted life years (DALYs) worldwide [2].

Situated in the tropics, Sri Lanka has an elevated risk of dengue endemics because mosquitoes thrive in warm, humid areas [3]. The severity of dengue in Sri Lanka has been increasing over the years. In 2010, 2012, 2014, 2016, and 2017, there were 34,188, 44,461, 47,502, 55,150, and 186,101 reported cases, respectively [4]. In the first half of 2017, the number of dengue cases was 4.3 times higher than the typical number of dengue cases for the same time period in previous years, leading to 215 deaths, with capital city Colombo having the most reported cases [5]. This may have resulted from the heavy rain and flooding that affected Sri Lanka, as well as the many construction developments that are underway in the rapidly changing urban landscape of Colombo.

A potential method to reduce dengue is to monitor the disease through surveillance by tracking the number of cases and investigating the outbreak source, and then tracing and eliminating the potential mosquito breeding grounds that could spread the disease [5]. However, health authorities are struggling to monitor and control the spread of the disease using their outdated and time-consuming paper-based systems [6].

To address this, Nanyang Technological University (NTU), Singapore, developed an integrated digital surveillance tool called Mo-Buzz to target dengue in the Colombo region. The application was made available to the local health authorities to integrate predictive surveillance, dengue hotspot mapping, civic engagement, and health education through social media. The health inspectors who would normally use paper-based forms to input potential breeding sites and paper maps to pinpoint dengue hotspots were able to report through the system, reducing reporting time considerably. A similar Mo-Buzz application was piloted with the general public to encourage reporting of potential breeding sites to the health authorities and educate them on how to prevent the spread of dengue. However, although Mo-Buzz was successful with Colombo health authorities, uptake of the application was not fully operationalized with the general public. The application had also become dated having been launched in 2013 and needed upgrading, both conceptually and technologically [7].

To make the Mo-Buzz applications contemporary and more effective, the research term organized a 5-day hackathon event, Epihack Sri Lanka, in Colombo in November 2017 with funding received from Skoll Global Threats Fund. Local and international information technology (IT) and health experts participated in Epihack Sri Lanka to improve capacities and capabilities of the existing applications. They collectively brainstormed, shared expert information and experiences, and guided each other in stimulating vibrant discussions to create prototyped digital tools to prevent the spread of dengue.

Typically, requirements to develop a health application are compiled by health experts and given to IT experts to develop independently with no additional input from the health professionals until an early prototype has been created. Due to the collaborative nature of Epihack, experts in both IT and health fields work together to mutually collaborate on the application, ensuring that requirements of the health experts are met in conjunction with the capabilities of the IT professionals. This allows for instant updates of any issues that arise.

The main aim of Epihack Sri Lanka was to develop a cutting-edge participatory reporting tool by building on the existing features of Mo-Buzz. The goal was to implement prevention strategies to battle dengue, bridge communication gaps in dengue control, and achieve effective communication between health authorities and the public.

Epihack Sri Lanka was the first of its kind in Sri Lanka and had the uniqueness of bringing together experts from different fields (health communication, doctors, information technology, etc) to develop a digital health solution in a collaborative manner rather than the usual competitive hackathon format. Little research has been done to observe what works and what can be improved in an event such as this, and, therefore, the objective of this paper was to examine the effectiveness and value of a 5-day Epihack workshop based on grounded theory approach, through field observations and qualitative interviews with the attendees of the event.

## Methods

### Data Collection: Sample and Procedures

This research was conducted among Epihack Sri Lanka attendees, and ethical approval was obtained from the university’s review board. Participants were observed in their area of work in ethnographic format and qualitatively interviewed in an informal manner to gather their experiences and opinion of the event during the 5-day period. The interviews were audio recorded to ensure descriptive validity, and observations and themes were noted.

### Participants

A total of 58 facilitators and participants (16 women) from 11 countries attended Epihack Sri Lanka; 22 attendees were health experts, and 36 were IT experts. The event consisted of participants from Sri Lanka, India, Pakistan, United States, Albania, Laos, Thailand, Singapore, Australia, Belgium, and Cambodia; 16% had taken part in a previous Epihack. Participants included 7 international health facilitators, 5 international and 8 local health participants, 4 international and 4 local IT facilitators, and 3 international and 27 local participants.

Participants included industry technological experts and epidemiologists from the epidemiological units in Colombo and local hospitals. In addition, participants consisted of public health inspectors; faculty and computer science students from NTU, University of Colombo School of Computing, and the Computer Society of Sri Lanka; and representatives from the Ministry of Health and Colombo Municipal Council.

### Interview Guide

A basic interview guide containing a set of open-ended questions was prepared in English so we could understand the interviewee, the type of work they are involved in, and how that work impacts (if it does) health communication. Respondents were asked to take part in the informal interview and after we received their consent, they were briefed about the interview and its purpose. Interviews were guided by the following themes: general questions about the interviewee and their work, the reason for their participation in such an event, and the benefits of having an event like this.

The average duration of conversations was 15 to 20 minutes; conversations were moderated by an experienced researcher. During the interview sessions, the researcher often summarized and clarified the answers that were vague. The researcher also encouraged the participants to verify the summarized statements before moving to the next question. Interview sessions were documented with audio recordings and were later transcribed.

### Qualitative Analysis

Transcripts were coded line by line based on grounded theory, a data analysis process that starts with the collection of data that is interpreted and developed into themes and explanatory theory. The analysis process consists of steps such as “coding data; developing, checking, and integrating theoretical categories; and writing analytic narratives throughout inquiry” [8]. Codes were further elaborated as new themes developed during the coding procedure. The key purpose of the research was to uncover crucial factors that work best and what needs improvement in an event such as this.

### Epihack Format

The 5-day Epihack event was held November 6-10, 2017, in Colombo. The experts took on the roles of facilitators and participants to share information, experiences, and guide teams during discussions to stimulate vibrant conversations and formulate best practices.

### Daily Schedule of Events

Day 1 of the event consisted of an introduction to the dengue problem within Sri Lanka and examples of various digital health surveillance solutions that have been implemented around the world to educate facilitators and participants about the challenges at hand. Talks covered topics such as global dengue prevention methods, current dengue issues facing Colombo, and existing applications such as Mo-Buzz. As the talks were predominantly health-based with focus on educating those who were not aware of the problems, a brainstorming session was organized—particularly for IT participants—to clarify information presented to them on the current dengue issues.

Day 2 entailed visiting dengue hotspots around Colombo. Three groups were each taken to two sites that have had high levels of dengue outbreaks such as construction sites, temples, parks, and schools to interview the locals and view the area. The aims of the field trips were to observe mosquito breeding sites and collect information from the site staff members or individuals to get a clearer picture of the dengue situation.

The goals of the field trips can be encapsulated into two main questions:

- What do they have (ie, what problems is the site currently facing and what current dengue prevention systems are in place)?
- What do they need (ie, what are the problems with the existing dengue systems and how feasible are their ideas to improve the situation)?

A mini discussion session was organized to share information gathered from the field trips, where each group presented their findings and brainstormed the issues and requirements at hand. Both health and IT experts contributed to the conversation to ensure that the requirements were valid and the technology was achievable. Possible work topics were also discussed during the session.

Days 3 and 4 consisted of amalgamating ideas and creating groups consisting of IT and health experts to develop the chosen work topics. First, facilitators met to discuss, categorize, and divide the project into 5 achievable subprojects or modules. Then one IT and one health facilitator were assigned to each group based on their expertise. This is different from usual hackathons where groups are typically created before ideas are defined due to the more collaborative nature of the event. The subprojects were then presented to the whole team, which was asked to select the group they wanted to work in, and ideas were explored further. Work topics that formed were all different facets of the same surveillance system to prevent similar ideas being redeveloped. Groups then began working on their ideas which included the following:

- Developing a database for public health officials
- Developing a framework for work management and visualization for public health officials and other stakeholders
- Creating a centralized database to consolidate all of the various information from different sources into one dashboard
- Creating educational content to educate construction workers, schools, and the general public

Each group consisted of approximately 12 members, of which approximately 8 people were IT experts. Throughout the two days, each group presented their ideas to the attendees at regular intervals to gather opinions, ideas, and potential issues from the other groups. The groups worked closely together throughout the process to ensure that each facet was developed in parallel with ideas that could be incorporated with the others.

Day 5 of the event involved each group presenting the developed ideas to invited guests and VIPs.

## Results

### Major Factors

**Figure 1:**
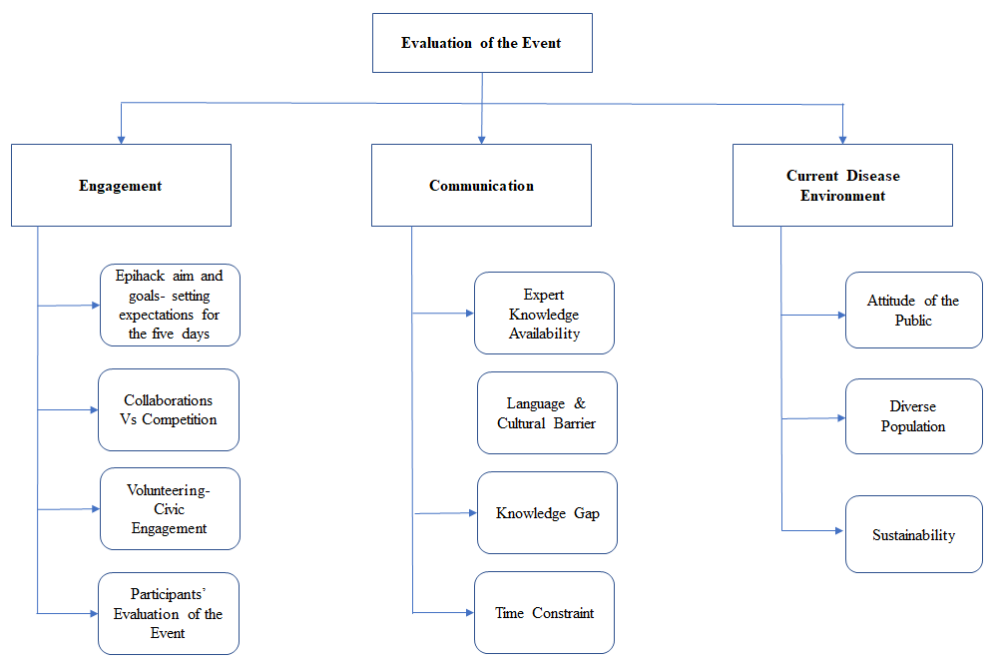
Themes and subthemes identified during the Epihack from qualitative interviews and observations.

The main aim of the study was to uncover the major factors that contributed to the effectiveness and value of the 5-day Epihack workshop. Three main themes were identified: engagement, communication, and environment (Figure 1).

### Engagement

#### Epihack Aim and Goals: Setting Expectations for the Five Days

Leading up to the event, a skilled Epihack organizer with experience in coordinating previous Epihacks assisted in the organization of this event. Facilitators were selected based on expertise, experience, and leadership qualities, with many facilitators having attended previous Epihacks. All facilitators were required to complete online training in Epihack facilitation before the event and attend pre-event meetings to ensure they were prepared to guide participants. During Epihack Sri Lanka, organizers and facilitators explained the aim and goals of the event to participants with examples from previous Epihacks conducted in other countries to clarify the nature of the event to participants.

One of the Epihack Sri Lanka organizers stated that the Epihack aimed to create a platform for effective communication and collaboration. The local health facilitators and health participants were encouraged to consider how to convey their needs in an understandable way to the IT experts to prevent a communication gap on the expected outcome.

A facilitator mentioned that “The future is not to work hard but to work smart,” and another facilitator added “The wheel has already been invented, but we want to make it faster.” They explained that the idea of an event such as Epihack Sri Lanka was to understand what had been previously done around the world, learn what had worked and had not worked, and borrow ideas and strategies used by previous teams rather than spending time on redeveloping the same ideas. Although the organizing team addressed the fact that it is difficult to come up with a perfect solution, they stressed that only by endeavoring and working together in this way to eradicate the disease can there be a step toward positive change: “It is one step closer to a perfect solution.”

Even though Epihack Sri Lanka was considered a hackathon, which traditionally focus on technology, it also welcomed nontechnology solutions. The primary goal was to create and brainstorm any ideas that could be possible solutions for reducing the spread of dengue.

#### Collaboration Versus Competition

The Epihack organizers and facilitators believe that Epihack works better if it is executed in a collaborative format instead of a competitive format. One of the facilitators suggested that competition and collaboration were needed in equal amounts for a workshop like this to work:

Collaboration and competition go hand in hand—they create team spirit and camaraderie. You need competition between the groups, so [that] the team gets more cohesion and self-identity to compete with the other team. It differentiates your team from the other. On the other hand, you are collaborating not on an individual level but at a team level; after you create the team identity, then at the next stage you start collaborating between the teams. It is a two-level logical approach. Collaboration and competition are not exclusive to one another. For this kind of event, you need both. You motivate the teams, one of the means is to say “Look, the other guys are doing better, we need to show [we can do better] ourselves.” It will put more morale into the team; it also makes the team more cohesive. So, people get to work with each other [everyone becomes a team player], as they have this common goal to compete with other teams. On the other hand, you are collaborating with other teams as each team depends on the other teams to get input and each team’s output is going as input to the other teams. So, you need to be very careful keeping a balance between the teams. The process is interconnected like an instrument [in an orchestra]. Each instrument plays its music, but all combined make the orchestra. You need to have the diligence of the orchestra.

Facilitator

One of the organizers mentioned that when she was getting participants to go and eat, one participant told her to give him five more minutes as he was finishing a task for another team so that they could start their work.

#### Volunteering: Civic Engagement

The Epihack core team believe that all the local and international facilitators and participants needed to volunteer their time to take part in the event:

People who come to do this volunteer their time to do so. We don’t pay anything because we need people who think it is for a good cause.

Organizer

One international facilitator mentioned that he came down to take part in the hackathon mainly because of his altruistic attitude. He felt he had done something good for society:

...it feels good, even though my feeling good goes along with creating something good for the society. On the other hand, I learn and develop a life experience which you don’t get in other settings.

International facilitator

He continued:

...participants and facilitators benefit from each other, the health participants get to learn technical stuff from the IT facilitators and participants, and, on the other hand, IT facilitators and participants get to learn health issues, possible solutions, etc. Additionally, participants get to learn from the local colleagues such as doctors. You understand the difficulties, you understand the setups, you understand the issues that are transposed to different sides of the world, [you may find] usable solutions: there are people everywhere.

International facilitator

Getting IT participants to volunteer for 5 days has been one of the toughest tasks in executing a collaborative Epihack event where there is no prize to be won in the end. Push notifications were sent out explaining the event details and inviting people working in IT sector to be part of the event. Even though the IT experts understood that it was an important cause and needed attention, they had busy schedules, previously committed deliveries, and deadlines. Potential participants were informed that Epihack Sri Lanka was an intensive 5-day workshop and that it was mandatory to participate in the full event, which put limits on the number of people who could take part in an event like this. However, it was the first event of its kind in Sri Lanka, making it a unique opportunity. Being a pioneering event, some IT participants could not gauge the event with the information provided at the time of registration. One of Epihack Sri Lanka organizers mentioned that getting IT experts to volunteer their time continued to be a major challenge in conducting Epihacks and therefore recruitment was ongoing until close to the event date. This was one of the reasons for the organizers not getting the complete profile and capabilities of the IT participants until the last minute, as they were the last group to be recruited to the event. However, when participants realized that their active participation and contribution could create a solution for the severe dengue problem in Sri Lanka, it boosted their involvement.

#### Participant Evaluation of the Event

After the event, attendees were briefly interviewed to gauge their opinion on the proceedings. Half of the participants thought that the event was an appropriate length and the majority of attendees were somewhat satisfied or extremely satisfied with the overall event:

I think that Epihack really showed attendees that many public health problems can be meaningfully engaged when there is a platform for interdisciplinary communication among professionals from multiple fields.

Health expert

However, some respondents believed the event could have been longer to support further development of the application, and 45% of respondents felt that the information given to them before their arrival at the event was not sufficient. Reasons for this include: “too short”, “didn’t have a much clear idea about what’s happening in 5 days. The target outcome and rough project ideas could have been shared with the event...with discussions” and there could have been “more explanation as to the agenda and details of what the hack entailed.”

The issues should be noted and improved for future Epihacks, with more information being given before the event to help attendees form a clearer picture of the event.

### Communication

#### Expert Knowledge Availability

The workshop brought experts from different parts of the world together for 5 days and created an environment where the international experts could assist and collaborate with the local team to come up with better solutions for solving a health problem. The international experts brought knowledge, expertise, and lessons learned from previous workshops and similar projects they had been part of in different countries. So, when ideas were proposed, they were able to give suggestions based on their previous experience of whether something would work or not. At the same time, experts also understood that each country had its own unique problems, but solutions could be found from other countries. “Adapt and apply” was one of techniques that was used here.

#### Language and Cultural Barriers

As the event consisted of an international group of people of varying ages and expertise, researchers identified certain gaps in communication during the event. During the first day of the event, language was one of the barriers as different people have different accents and ways of communicating their ideas. As the days progressed, participants got more acquainted with each other and this barrier drastically dropped. Fortunately, English is an official language of Sri Lanka and is spoken well by the majority of local people; therefore, it could be used as the working language for the event. This has not been the case in other Epihacks, where working in English was difficult as locals tended to revert to their local language, which made it difficult to work with international participants. Language was not the only barrier identified:

Some people are shy, everyone has an opinion but when you mix the group, they don’t want to tell, or sometimes they don’t get to tell. When there are many high-ranking people who attend, local junior people don’t want to speak.

Facilitator

As day 2 progressed, the imagined power-distance dropped, and participants and facilitators started talking to each other more freely. Even the student participants who had never worked before were working well with professionals and lecturers.

The research team noticed, however, that an informal hierarchy was perceived or practiced by the participants with white males at the top, followed by white females, local males, and females in that order. This perceived hierarchy and small number of female IT professionals on the team seemed to restrict them putting forth their viewpoints to the entire group.

Sri Lanka has a laid-back culture and people from various countries may have different working styles (preference to work alone, work in the morning or late at night, etc). As this was an event that ran on tight schedule, participants found it a bit rushed during the first couple of days. But they managed to put aside their preferences and come in on time as the event demanded their commitment.

#### Knowledge Gap

In an ideal world, the client would know what they want. However, in Epihack Sri Lanka the health team knew their disease burden and problem, but they didn’t have the solution to help them fix the disease situation. This is where an event like Epihack can be a platform for health authorities to collaborate with international experts to discuss ideas and solutions from around the world and see if any could be adapted and applied in Sri Lanka. The event also brings together local health experts who do not usually get the time to talk to each other about the health and disease problems they handle. Officers from different areas of Colombo might face different problems (eg, dealing with more wealthy or commercial areas). While interacting with the local health experts, the research team also noticed that some offices were well equipped, but some others were not as well maintained.

There is another kind of knowledge gap that exists between the IT experts and health experts. The workshop was planned in such a way that the health facilitators would lead the first 2 days of the discussion, informing the IT team of the problems and challenges, and clarifying any queries from the IT team. It was mentioned that 2 days is very small amount of time to understand all the procedures and workflows; at the same time, the 2 days were conducted mostly in lecture style rather than group discussion format, which some of the IT people found overwhelming. However, the main goal was to let the developing teams understand critical problems and find ideas that can help to ease the disease burden. The whole idea of Epihack is to bring together multidisciplinary teams who never usually interact so that different angles can be used to see the big picture.

One of the IT facilitators mentioned that he preferred to use “bottom up approach, not top down approach” to bridge requirement and knowledge gaps. He explained further that this approach helps them to find the missing element in the whole system. Once they know the missing elements, it is easier to put the pieces together. The method helps them to formulate an action-oriented plan.

#### Time Constraint

After 2 days of discussion and brainstorming sessions between IT and health professionals, the IT facilitators and participants had 48 hours to create a tangible prototype of the proposed solution. The amount of time was so limited that the focus was to get all the ideas in and create a quick prototype, which could later be expanded to a workable solution. IT experts opined that it would take another 4 to 6 months of work for the prototype to be converted into a full-fledged working application. However, the health team experts were impressed by the amazing amount of effort that the IT team had put together in just 2 days in creating a prototype:

I would add an extra day and night of hacking in order to allow our participants more time in polishing up the prototypes developed.

Participant

### Current Disease Environment

A doctor explained the current disease environment with the following quote.:

Dengue is a complicated problem, we can’t pin point to what are the things we want.

Local health facilitator, doctor

#### Public Attitude

Currently, even if the public is aware of the dengue situation and how to reduce the spread, they do not necessarily follow dengue prevention methods. Civic engagement in preventing the spread would reduce the amount of time and work required from public health inspectors (PHIs). Presently, the PHIs must go from house to house doing inspections. PHIs are also bombarded with other problems such as garbage collection, which should be handled by the sanitation department. They are put in a situation where they feel that they are required to follow up on all the complaints as they need to keep a good relationship and good reputation with the public. Solutions suggested by the participants were to get the public involved in the process, make them feel empowered, and show them the value of their actions to make them feel like they are part of the dengue control activity group.

#### Diverse Population

As the public consists of a diverse group of people, such as people from different education backgrounds and migrant workers from China who don’t follow the Sinhala language but work in highly dengue prone areas, it is important to think about the target audience, the purpose of the application, and the ways to get people to use the application. As one of the local doctors said, many members of the public don’t even know “[basic] information and knowledge, like what the normal temperature is or what the color of blood is,” suggesting that people in the capital belong to diverse population.

#### Sustainability

A few types of sustainability issues were identified during the workshop that will need local support and groundwork to keep the project running. One of the health experts mentioned that local stakeholders must be the ones to sustain the product as they know their environment:

The application is not the challenge; it is the social and government support that will be critical for a project like this.

Health expert

They believe that this project should be outlined as a social responsibility project, and the work needs to be continued after the workshop:

How to get the tools is difficult, but how to maintain and continue working with the tool is most difficult.

Health expert

Factors such as manpower and finding skilled collaborations also affect sustainability.

After the Epihack, another team will have to develop this prototype into a workable solution, and there needs to be continuous communication and collaboration between the hackathon participants and the solution developers for the process to be smooth. During the last day, the work done and ideas created during the hackathon were showcased to stakeholders, government representatives, and the media. Support from key stakeholders would go a long way in fruitful completion of the project.

Another important thing to consider is to get public attention and get them to use the app to report mosquito breeding sites. Campaigns, celebrity endorsements, gamifications, and social media presences were a few of the ideas that were developed as part of the brainstorming process.

## Discussion

### Principal Findings

An Epihack is a civic engagement–based health hackathon that brings different field experts and participants together from all over the world to work on health problems. Previous Epihacks focused on several health problems usually led by health experts such as epidemiologists, and the solutions created have been developed further and put to use to control the disease burden [9]. The current Epihack was the first ever Epihack to be led by health communication experts.

The main aim of Epihack Sri Lanka was to create a platform to reduce the health burden of an increasingly prevalent infectious disease through multidisciplinary teamwork. This was attempted through proper training and pre-event meetings to ensure that the facilitators were prepared to guide the participants. Epihack is unique in its format, as it is a collaborative event and not a competitive one. There was no winning team per se; the groups assisted one another to make the workshop successful. The culmination of ideas led to each group developing a facet of a larger digital health system. We observed many advantages to this type of hackathon format. It creates an environment where groups feel that they can share ideas and develop them further with each other rather than feel the need to hide their concepts from each other. Each group was able to work with others on different parts of a single system rather than producing and developing similar overlapping ideas. The realization that other teams were waiting for their inputs made each team speed up their work. It was clear that the concept of team-bonding was effective, because they were working toward one common goal rather than individual goals. The collaborative format may also be a potential method for other hackathons. In the future, this experience may lead to more hackathons working in this way, leading to a greater variety of ideas being produced.

Getting IT participants to volunteer was one of the major challenges that the organizers faced before the event. Better planning before the event can help in handling this challenge. From participants’ feedback, we learned that more information needs to be given to the IT developers before the event so they can prepare better for the event.

The broad range of expert knowledge availability from different disciplines is one of the major advantages of the event and should be used to the maximum for idea development and implementation. However, it is crucial to understanding the level of knowledge of the attendees for a collaborative event that brings participants from different fields and expertise. Language and cultural barriers are also facets that need to be taken into consideration while preparing for an event, especially when the event consists of participants from diverse international backgrounds. This is to reduce communication issues and increase the effectiveness of the event. As the workshop is tightly planned, the organizers should also be aware of time constraints for developing and incorporating all ideas during the workshop and they should make sure attendees are aware of the limitation in development time.

Locals need to be completely invested in the event as they will be the key players after the Epihack is executed. During Epihack Sri Lanka, international participants were able to encourage the local participants with their ideas and experiences. After the end of 5 days, local participants from Sri Lanka created a Facebook page to keep in touch with all the other participants. This shows that the collectivist team mentality created a bond over the event period.

During disease outbreaks, when there is a time crunch, stakeholders may rush to produce an application or a solution in very little time and without much knowledge. This makes the application a part of a checklist rather than being a truly optimal solution for society’s problem. Epihack could be a potential way to help a community to get ideas and solutions within a small period of time, as it focuses on the creation of ideas to solve the disease problem rather than just quickly creating a prototype without much input from experts.

Sustainability is another key element that needs to be addressed as it will affect the work to be done after the Epihack. There are two kinds of sustainability concerns. First, the stakeholders need to get involved in the project to get proper funding and ground support. Second, public participation will be imperative when the mobile application goes public. So the team should be well prepared to motivate the public for continuous use of the application. In this paper, we identified the list of items that worked best as well as the items that could be improved. The list and our recommendations are provided in Multimedia Appendix 1.

### Limitations

The total number of attendees for the event was less than 60, making this a study with a small participant pool. Future studies need to be planned to build on this knowledge from the workshop. By observing and interviewing more attendees of Epihack, a pattern of what works best can be developed and precisely streamlined.

### Conclusion

Events such as Epihack are a great way to bridge gaps between different fields of work. Through qualitative interviews, the process was found to be largely positive and fruitful in the development of an integrated digital surveillance application. The application was developed by the local IT team from the system prototype created during the event and finished in Dec 2018. Frequent discussions with the stakeholders will ensure proper uptake of the application. Pilot testing and usability studies are scheduled to take place after the development phase. Future events such as these should focus on engaging attendees and being aware of communication issues within the workshop environment. The effectiveness of controlling dengue is linked to the number of dengue cases. There needs to be a long-term full commitment by the decision makers that can control and ensure the proper routine work flow of hotspot identification, insecticide use, fogging activities, patient management, enforcement of hotspot penalties, public cooperation, and disease surveillance with the assistance of technologies. From the success of this event, future hackathons may benefit by following this model.

## Appendix

Multimedia Appendix 1 Recommendations.

## Abbreviations

DALY: disability-adjusted life year
IT: information technology
NTU: Nanyang Technological University
PHI: public health inspector
