# Supplementary material for: Developing a Digital Solution for Dengue Through Epihack: Qualitative Evaluation Study of a Five-Day Health Hackathon in Sri Lanka
Source: JMIR Form Res. 2019 Aug 29;3(3):e11555. doi: 10.2196/11555 (PMC6740162; doi:10.2196/11555)
Supplement: Multimedia Appendix 1 [file formative_v3i3e11555_app1.pptx]

## Slide 1
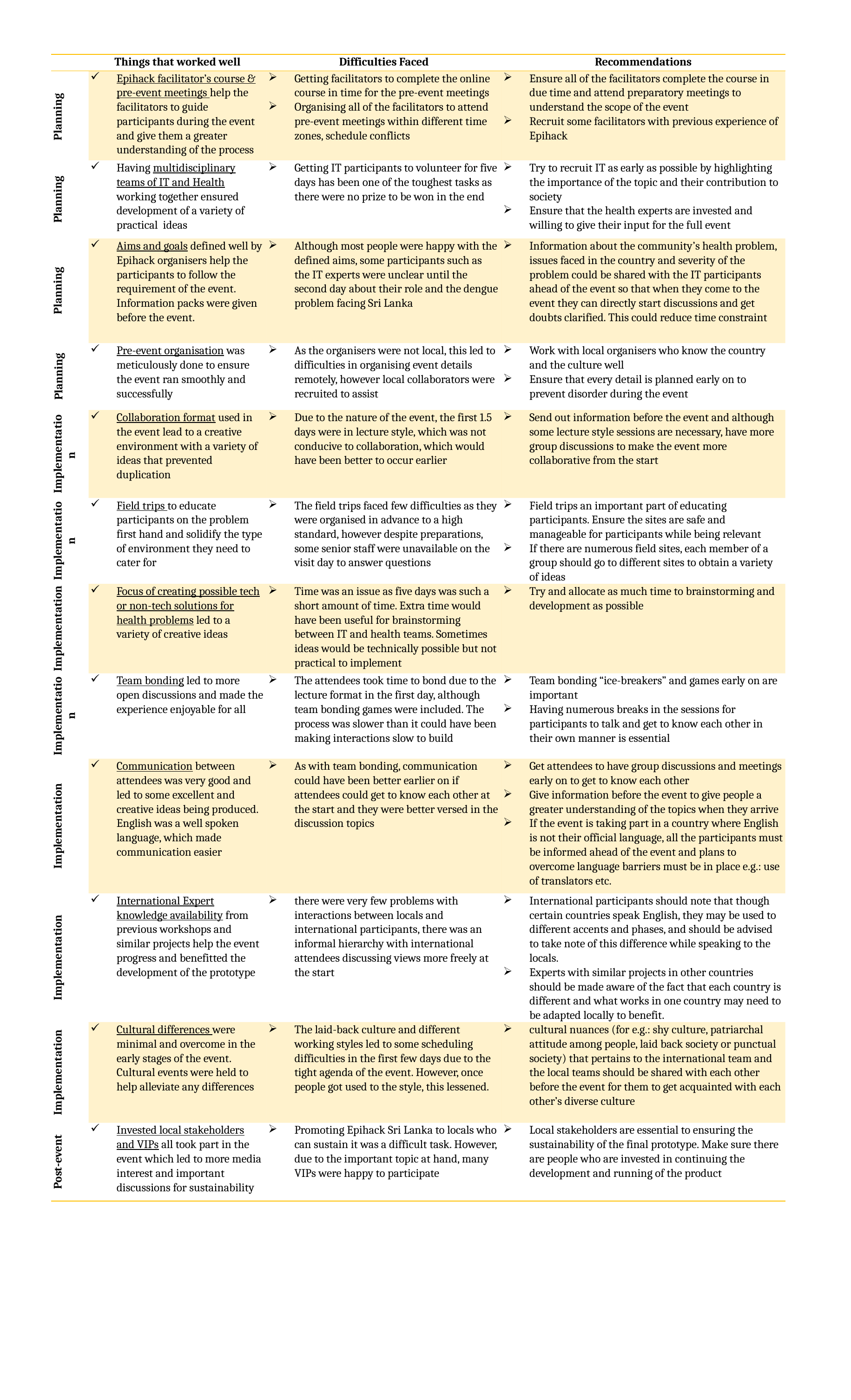

| | Things that worked well | Difficulties Faced | Recommendations |
| --- | --- | --- | --- |
| Planning | Epihack facilitator’s course & pre-event meetings help the facilitators to guide participants during the event and give them a greater understanding of the process | Getting facilitators to complete the online course in time for the pre-event meetings Organising all of the facilitators to attend pre-event meetings within different time zones, schedule conflicts | Ensure all of the facilitators complete the course in due time and attend preparatory meetings to understand the scope of the event Recruit some facilitators with previous experience of Epihack |
| Planning | Having multidisciplinary teams of IT and Health working together ensured development of a variety of practical ideas | Getting IT participants to volunteer for five days has been one of the toughest tasks as there were no prize to be won in the end | Try to recruit IT as early as possible by highlighting the importance of the topic and their contribution to society Ensure that the health experts are invested and willing to give their input for the full event |
| Planning | Aims and goals defined well by Epihack organisers help the participants to follow the requirement of the event. Information packs were given before the event. | Although most people were happy with the defined aims, some participants such as the IT experts were unclear until the second day about their role and the dengue problem facing Sri Lanka | Information about the community’s health problem, issues faced in the country and severity of the problem could be shared with the IT participants ahead of the event so that when they come to the event they can directly start discussions and get doubts clarified. This could reduce time constraint |
| Planning | Pre-event organisation was meticulously done to ensure the event ran smoothly and successfully | As the organisers were not local, this led to difficulties in organising event details remotely, however local collaborators were recruited to assist | Work with local organisers who know the country and the culture well Ensure that every detail is planned early on to prevent disorder during the event |
| Implementation | Collaboration format used in the event lead to a creative environment with a variety of ideas that prevented duplication | Due to the nature of the event, the first 1.5 days were in lecture style, which was not conducive to collaboration, which would have been better to occur earlier | Send out information before the event and although some lecture style sessions are necessary, have more group discussions to make the event more collaborative from the start |
| Implementation | Field trips to educate participants on the problem first hand and solidify the type of environment they need to cater for | The field trips faced few difficulties as they were organised in advance to a high standard, however despite preparations, some senior staff were unavailable on the visit day to answer questions | Field trips an important part of educating participants. Ensure the sites are safe and manageable for participants while being relevant If there are numerous field sites, each member of a group should go to different sites to obtain a variety of ideas |
| Implementation | Focus of creating possible tech or non-tech solutions for health problems led to a variety of creative ideas | Time was an issue as five days was such a short amount of time. Extra time would have been useful for brainstorming between IT and health teams. Sometimes ideas would be technically possible but not practical to implement | Try and allocate as much time to brainstorming and development as possible |
| Implementation | Team bonding led to more open discussions and made the experience enjoyable for all | The attendees took time to bond due to the lecture format in the first day, although team bonding games were included. The process was slower than it could have been making interactions slow to build | Team bonding “ice-breakers” and games early on are important Having numerous breaks in the sessions for participants to talk and get to know each other in their own manner is essential |
| Implementation | Communication between attendees was very good and led to some excellent and creative ideas being produced. English was a well spoken language, which made communication easier | As with team bonding, communication could have been better earlier on if attendees could get to know each other at the start and they were better versed in the discussion topics | Get attendees to have group discussions and meetings early on to get to know each other Give information before the event to give people a greater understanding of the topics when they arrive If the event is taking part in a country where English is not their official language, all the participants must be informed ahead of the event and plans to overcome language barriers must be in place e.g.: use of translators etc. |
| Implementation | International Expert knowledge availability from previous workshops and similar projects help the event progress and benefitted the development of the prototype | there were very few problems with interactions between locals and international participants, there was an informal hierarchy with international attendees discussing views more freely at the start | International participants should note that though certain countries speak English, they may be used to different accents and phases, and should be advised to take note of this difference while speaking to the locals. Experts with similar projects in other countries should be made aware of the fact that each country is different and what works in one country may need to be adapted locally to benefit. |
| Implementation | Cultural differences were minimal and overcome in the early stages of the event. Cultural events were held to help alleviate any differences | The laid-back culture and different working styles led to some scheduling difficulties in the first few days due to the tight agenda of the event. However, once people got used to the style, this lessened. | cultural nuances (for e.g.: shy culture, patriarchal attitude among people, laid back society or punctual society) that pertains to the international team and the local teams should be shared with each other before the event for them to get acquainted with each other’s diverse culture |
| Post-event | Invested local stakeholders and VIPs all took part in the event which led to more media interest and important discussions for sustainability | Promoting Epihack Sri Lanka to locals who can sustain it was a difficult task. However, due to the important topic at hand, many VIPs were happy to participate | Local stakeholders are essential to ensuring the sustainability of the final prototype. Make sure there are people who are invested in continuing the development and running of the product |
